# Fistulising skin metastases in Crohn’s disease: a case report and review of the literature

**DOI:** 10.1186/s13256-024-04569-1

**Published:** 2024-05-19

**Authors:** Tanja Elger, Johanna Loibl, Christa Buechler, Sebastian Haferkamp, Jens Werner, Konstantin Drexler, Ulrich Hohenleutner, Karsten Guelow, Claudia Kunst, Arne Kandulski, Pia Goeggelmann, Martina Mueller, Hauke Christian Tews

**Affiliations:** 1 Department of Internal Medicine I, Gastroenterology, Hepatology, Endocrinology, Rheumatology and Infectious Diseases, University Hospital Regensburg, Franz-Josef-Strauß-Allee 11, 93053 Regensburg, Germany; 2 Department of Visceral Surgery, University Hospital Regensburg, Regensburg, Germany; 3 Department of Dermatology, University Hospital Regensburg, Regensburg, Germany

**Keywords:** Metastatic Crohn’s disease, Skin fistulae, Ustekinumab, Inflammatory bowel disease, Extraintestinal manifestation

## Abstract

**Background:**

Metastatic Crohn’s disease is a rare disorder characterized by various granulomatous skin lesions that occur independently of gastrointestinal tract involvement. However, currently there is no standardized care or specific treatment. Therapeutic approaches include immunosuppressive agents, such as corticosteroids, azathioprine, and monoclonal antibodies targeting inflammatory cytokines like tumor necrosis factor (TNF).

**Case presentation:**

We present a case of a 29-year-old western European woman with significant blind ending abdominal subcutaneous fistulas and abscesses, who sought evaluation in the dermatology department. Histological examination revealed multiple epithelioid cell granulomas. There was no evidence of infectious or rheumatologic diseases such as sarcoidosis. The tentative diagnosis was metastatic Crohn’s disease, which was not related to an intestinal manifestation of the disease. The patient responded to infliximab but had to discontinue it due to an allergic reaction. Subsequent adalimumab treatment failed to induce clinical remission; thus, therapy was switched to ustekinumab, resulting in a positive response. Written informed consent for publication of their clinical details and clinical images was obtained from the patient.

For our study more than 1600 publications were screened for cases of metastatic Crohn’s disease on PubMed database. 59 case reports with 171 patients were included in the analysis and evaluated for localization, diagnostic and therapeutic approaches, and complications and were summarized in this review.

**Conclusion:**

The successful ustekinumab treatment of a patient with metastatic Crohn's disease underscores the potential of this minimally investigated therapeutic option, highlighting the need for future treatment guidelines given the increasing prevalence of such cases.

## Background

Metastatic Crohn`s disease is a rare disease that primarily affects patients diagnosed with inflammatory bowel diseases (IBD). In rare cases, cutaneous manifestations precede gastrointestinal involvement. It most commonly occurs in the genital region, but can also affect every other part of the body. The lesions present as plaques, ulcers, fissures or papules [1] and, in rare cases, as fistulas. They are often asymptomatic but may also cause pain or itching. Despite the misleading wording, metastatic Crohn’s disease is not considered an oncologic entity. Instead, the term “metastatic” refers to the involvement of sites with no physical connection to the GI-tract. Metastatic Crohn’s disease must not be mixed up with extraintestinal manifestations of Crohn´s disease. Whereas extraintestinal manifestations such as pyoderma gangraenosum and erythema nodosum represent distinct immunologic phenomena, metastatic Crohn’s disease exhibits the same histological findings as intestinal Crohn’s disease, but on other organ sites.

Usually, a biopsy from the involved site is required for the diagnosis of metastatic Crohn’s disease. Histological findings show non-caseating, sarcoid-like granulomas, Langerhans giant cells and foreign body giant cells surrounded by inflammatory histiocytes, plasma cells and lymphocytes [2]. For a confirmed diagnosis of metastatic Crohn’s disease, other causes for granulomatous disorders have to be excluded, especially cutaneous sarcoidosis, tuberculosis, syphilis, mycobacterial infections, actinomycosis, deep fungal infections, lymphogranuloma venereum and granuloma inguinale. Also non-granulomatous skin lesions such as hidradenitis suppurativa, pyoderma gangrenosum, impetigo, erythema nodosum, factitial dermatitis from factitial injection of foreign substances, schistosomiasis, chronic lymphedema resulting from obstruction, erysipelas, chronic cellulitis and foreign body reaction need to be ruled out [3].

To objectively assess intestinal involvement, endoscopy of the upper and lower GI tract along with an abdominal magnetic resonance imaging (MRI) scan should be performed.

Currently, there is no standardized treatment of metastatic Crohn’s disease and no German or European guideline, especially for cases without GI involvement. Only individual case reports exist regarding the therapeutic use of approved medication for intestinal Crohn’s disease in metastatic conditions, including steroids, anti-TNF antibodies, azathioprine, and antibiotic therapy [4–7].

Ustekinumab is a human monoclonal IgG1κ-antibody, which binds specifically to the p40-subunit of interleucin 12 (IL-12) and interleucin 23 (IL-23). The bioactivity of IL-12 and IL-23 is inhibited by ustekinumab by preventing the p40 subunit from binding to IL-12Rß1-receptorprotein on the surface of immune cells. It is assumed that hereby the cytokine pathways of Th1- and Th17-cells are interrupted, which both play an important role in the pathogenesis of Crohn’s disease [8].

We here report a case of metastatic Crohn’s disease successfully treated with ustekinumab at our university hospital and provide a literature review on the current therapeutic options for metastatic Crohn’s disease.

## Case report

In 2020, a 29-year-old western European woman presented to the dermatology department of our university hospital with pronounced abdominal blind-ending fistulas. The initial patient contact was documented in 2019 when she sought care at the emergency department with multiple recurrent abscesses of the abdominal skin that first appeared after a tick bite a few weeks prior.

**Fig. 1:**
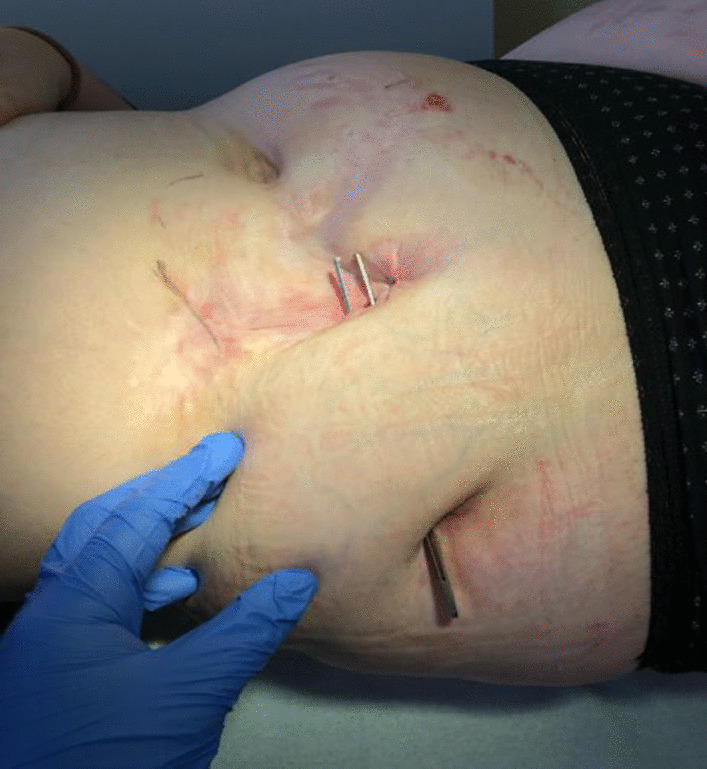
Large subcutaneous fistula, 2020

**Fig. 2:**
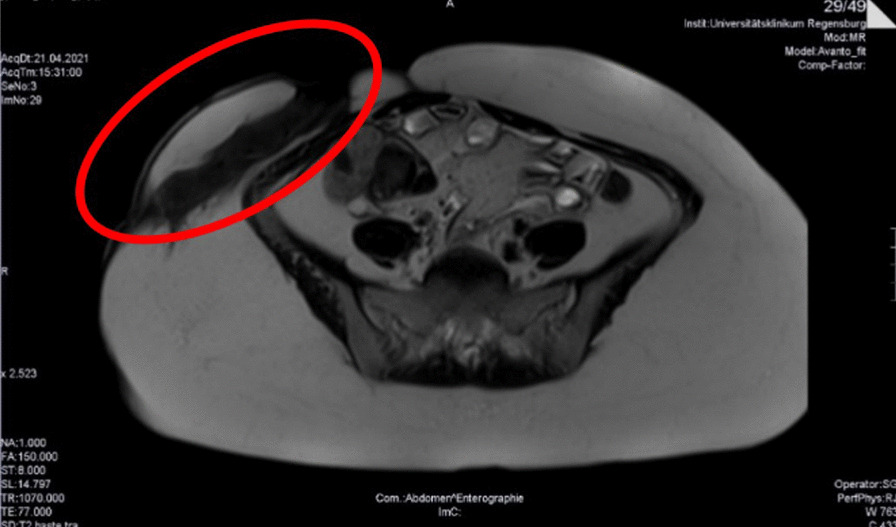
MRI enterography of the large subcutaneous abdominal fistula (red circle), April 2021

Between January 2019 and July 2020, multiple incisions of recurrent abscesses were performed in combination with antibiotic treatment. However, complete healing was not achieved. Instead, the abscess cavities expanded beneath the skin, subsequently forming a system of connected fistulas (Fig. 1), as visualized in MRI enterography (Fig. 2).

When the patient presented to the dermatology department in 2020, the fistulas showed active inflammation with secretion and were almost 2 cm in diameter. In addition, a blind ending subcutaneous fistula of the thigh could be detected, which also showed active inflammation and secretion.

**Fig. 3:**
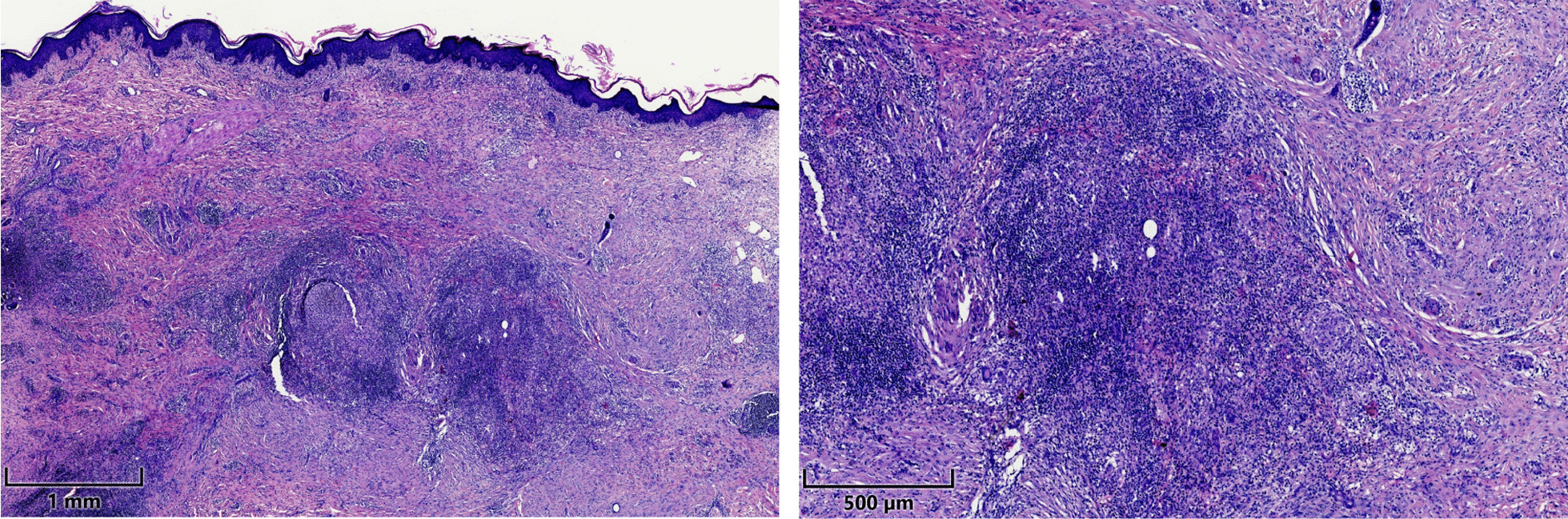
Histologic examination of a fistula biopsy showing granulomas with histiocytes and multinuclear giant cells, July 2020

Histologic examination revealed a granulomatous infiltrate with histiocytes and multinuclear giant cells, forming granulomas. In addition, lymphocytes, plasma cells, neutrophils, and eosinophils, and a granulomatous perivasculitis were observed (Fig. 3). Differential diagnoses of granulomatous diseases such as sarcoidosis, tuberculosis and immunodeficiencies were ruled out, confirming the diagnosis of metastatic Crohn’s disease.

To investigate intestinal involvement, abdominal MRI, gastroscopy, and colonoscopy were performed. However, these examinations did not reveal any gastrointestinal involvement at this time. Moreover, fecal calprotectin levels were normal. There was no history of inflammatory bowel disease in the patient’s family.

Antibiotic therapy had failed in the past, but the Patient responded well to prednisolone. As maintenance therapy for metastatic Crohn’s disease, the patient initially received infliximab at a dosage of 5 mg/kg. The patient responded well to this therapy, leading to the cessation of fluid secretion by the fistulas. However, an allergic reaction with dyspnea and rash occurred after the 10th dose of infliximab in October 2021, necessitating the discontinuation of treatment.

To evaluate further treatment options, the patient was referred to the department of Gastroenterology in November 2021. Meanwhile, she had developed diarrhea and abdominal pain. MRI revealed a mild ileitis, which, however, could not be validated by colonoscopy.

Therapy was switched to adalimumab in December 2021, to which the patient did not respond. Instead, a new fistula ostium developed.

Despite the lack of evidence for ustekinumab therapy in metastatic Crohn’s disease, and given its established efficacy only for intestinal manifestation, treatment with ustekinumab was initiated in May 2022 with an initial dose of 390 mg intravenous, followed by 90 mg subcutaneous every 8 weeks. This decision was based on the suspicion that the inflammatory processes in the abdominal fistula mirrored those seen in intestinal inflammation. The patient responded well and inflammation decreased within a few weeks. However, fistulas persisted, albeit with reduced secretion.

In June 2022, the blind-ending subcutaneous fistula of the thigh could be successfully treated by surgery after the active inflammation resolved. In April 2023, the abdominal fistula also showed no remaining inflammation, so a complete excision of the abdominal fistulas was performed. At the patients last visit in August 2023, no new fistula or abscesses were detected, but ustekinumab was continued due to the long and complicated clinical history. Written informed consent for publication of their clinical details and clinical images was obtained from the patient.

## Review of the current literature

### Material and methods

A data base literature search was performed using the keywords “metastatic” and “Crohn’s” and “disease”. 1,875 reports published from January 2012 to November 2023 were found. The number of papers meeting the search criteria steadily increased, highlighting the clinical relevance of this topic.

So far, 59 case reports including 171 patients and 12 reviews about clinical presentation, diagnostic approach and therapeutic options have been published. However, no statistical analysis of patient characteristics and treatments is available. Therefore, our aim was to objectively assess these items. Moreover, we intended to discuss ustekinumab as a novel but successful therapeutic approach for metastatic Crohn’s disease.

### Results

Of the patients with metastatic Crohn’s disease, 74% were female, and 38% of the cases involved individuals under 18 years old. From this cohort, we assessed diagnostic and therapeutic approaches for metastatic Crohn’s disease.

80% of the patients were diagnosed with intestinal Crohn’s disease before or at the time skin lesions appearance, while 10% exhibited gastrointestinal symptoms such as diarrhea or abdominal pain without IBD [9–19]. 7% had isolated extraintestinal manifestations as illustrated in our presented case. Fistulas were described in 13% of all cases [4, 20–22], highlighting the rarity of this condition. However, fistula presence or absence was not explicitly mentioned in 58% of all published cases.

At the time of diagnosing metastatic Crohn’s disease, usually skin biopsies are taken and examined. Granulomas were present in 58% of all cases [3–7, 10–17, 19–46], with some studies not commenting on histological findings.

 (Fig. 4).

**Fig. 4:**
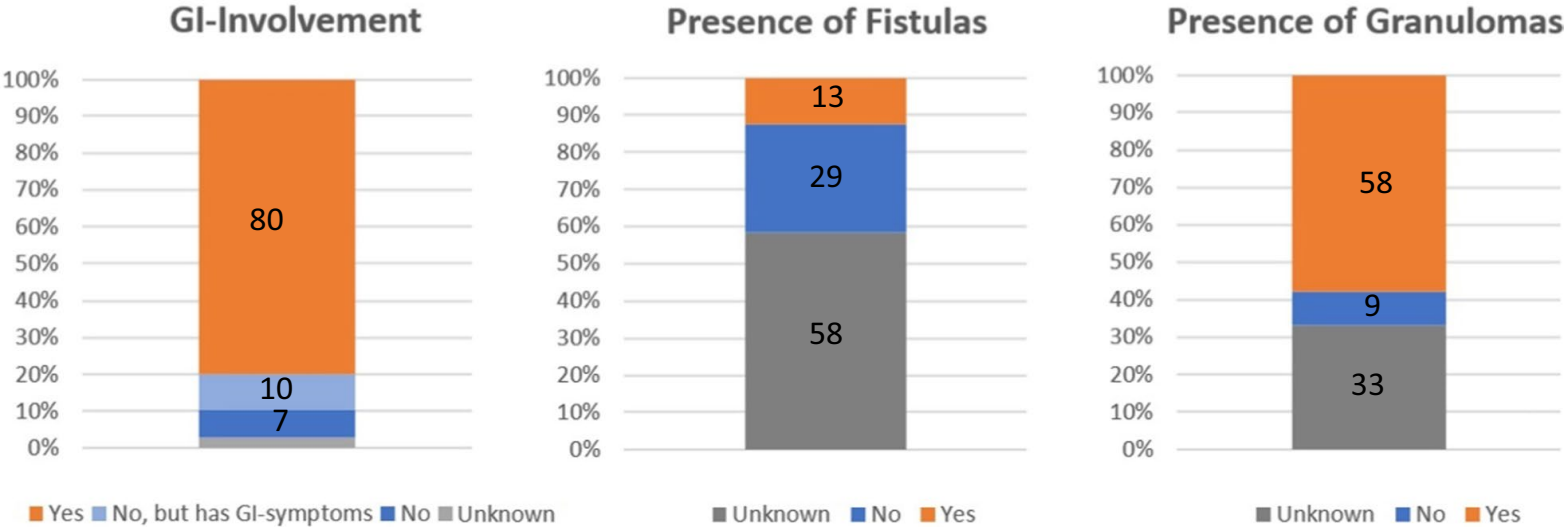
Proportion of patients with GI-Involvement, granuloma and fistula (*n* = 171)

**Fig. 5:**
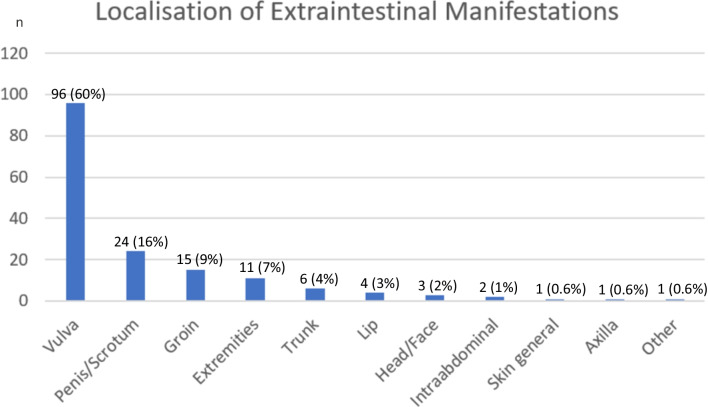
Localisation of lesions in patients with metastatic Crohn’s disease (*n* = 159)

The most common localization of metastatic Crohn’s disease was the genital region, including groin, vulva and penis/scrotum (Fig. 5). Notably, our case report highlights that metastatic Crohn’s disease can affect almost every part of the body. This is in accordance with other reports describing affected skin at various sites of the body including extremities, trunk or head/face [4, 5, 16, 22, 25, 30–32, 36–38, 41, 44, 47, 48].

Less common sites of involvement included intraabdominal abscesses [31, 49] and necrotizing endocarditis of the aortic valve with granuloma formation [26]. Some patients also exhibited lesions on more than one site. They were counted for each site in Fig. 5. For 12 patients, the site of involvement was not mentioned.

**Fig. 6:**
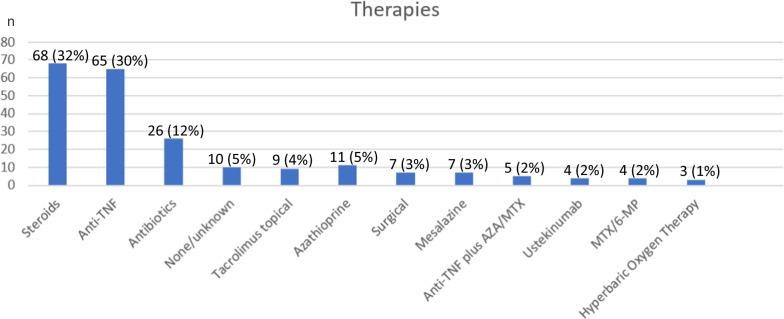
Therapeutic approaches for metastatic Crohn’s disease (*n* = 219)

There are no general recommendations for the treatment of metastatic Crohn’s disease, but similar pathophysiological processes are suspected. Hence, established therapeutic regimens for the treatment of intestinal Crohn’s disease are employed. The most common therapeutic approach involves corticosteroid admission (Figure 6), often resulting in a lesion reduction. Anti-TNF, especially infliximab, was effective and utilized in 41.6% of cases. Some patients received multiple therapies and were counted accordingly (Fig. 6).

Steroid treatment led to remission or improvement in 30.8% of all described cases (Table 1). Excluding unknown cases, steroids induced remission (17.2%) or improvement (55.2%) in 72.4% of patients.

**Table 1:** Response to therapeutic options

Response	None, *n* (%)	Remission *n* (%)	Improvement *n* (%)	Worsening *n* (%)	Unknown *n* (%)	Total *n* (%)
Steroids	6 (9.1)	5 (7.6)	16 (24.2)	2 (3.0)	37 (56.1)	66 (100)
Anti-TNF	5 (7.8)	2 (3.1)	8 (12.5)	0 (0)	49 (76.6)	64 (100)
Antibiotics	5 (22.7)	4 (18.2)	8 (36.4)	1 (4.5)	4 (18.2)	22 (100)
None/unknown	0 (0)	0 (0)	0 (0)	0 (0)	10 (100)	10 (100)
Tacrolimus topical	3 (33.3)	0 (0)	5 (55.6)	0 (0)	1 (11.1)	9 (100)
Azathioprine	0 (0)	3 (33.3)	4 (44.4)	0 (0)	2 (22.2)	9 (100)
Surgical	2 (28.6)	1 (14.3)	1 (14.3)	0 (0)	3 (42.9)	7 (100)
Mesalazine	0 (0)	2 (28.6)	2 (28.6)	1 (14.3)	2 (28.6)	7 (100)
Anti-TNF plus AZA/MTX	1 (25.0)	0 (0)	2 (50.0)	1 (25.0)	0 (0)	4 (100)
Ustekinumab	0 (0)	2 (66.7)	1 (33.3)	0 (0)	0 (0)	3 (100)
MTX/6-MP	1 (25.0)	1 (25.0)	0 (0)	0 (0)	2 (50.0)	4 (100)
Hyperbaric Oxygen Therapy	1 (33.3)	1 (33.3)	1 (33.3)	0 (0)	0 (0)	3 (100)

N, number of patients; %, percentage of response of all patients [3–7, 9–63]

Anti-TNF, especially infliximab, less common also adalimumab and certolizumab, only had an effect (improvement or remission) in 15.6% of the cases. However, excluding unknown cases, remission was induced in 13.3% and improvement in 53.3% of patients (Table 1).

Antibiotics, azathioprine and topical tacrolimus also showed positive effects, although azathioprine did not induce remission in any patient. Data regarding surgical interventions, mesalazine, anti-TNF plus azathioprine, MTX/6-MP, ustekinumab, and hyperbaric oxygen therapy are limited and inconclusive based on the literature. Notably and in line with the presented case, two out of three patients achieved remission after treatment with ustekinumab.

## Discussion

Our examination summarizes patient characteristics in metastatic Crohn’s disease and outlines therapeutic options based on the presented case. However, there are some limitations.

We established the diagnosis of metastatic Crohn’s diseases based on clinical and histological findings. Other granulomatous diseases such as sarcoidosis, tuberculosis and immunodeficiencies were ruled out, and histological findings were typical for Crohn’s lesions despite there was no intestinal involvement at the time of diagnosis. However, there is no possibility of completely ruling out any other underlying immunological condition causing similar symptoms. The analyzed case reports mostly also based their diagnosis on histological findings fitting Crohn’s criteria, and often on a known underlying intestinal Crohn’s disease, but in some publications, diagnostic criteria were not discussed in detail.

Our analysis is a summary of published cases and no randomized trial, making it impossible to compare the efficiencies of different therapies. This article outlines various treatment approaches and their success rates according to literature data. However, assessing the patient's condition before and after treatment from literature data can be challenging.

Several patients responded well to antibiotic treatment (see Table 1). This was also effective in some patients with GI involvement. Nevertheless, it remains unclear if lesions described as metastatic Crohn’s disease are infectious or a manifestation of Crohn’s disease.

Despite these limitations, treatments for metastatic Crohn`s disease could be evaluated. The most effective treatments, according to the current literature, include steroids and anti-TNF-antibodies. Despite the small number of cases, also azathioprine showed good clinical results. Mesalazine also appeared to positively impact skin lesions in patients with metastatic Crohn’s disease (see Table 1).

Conclusively, mesalazine, azathioprine, steroids, and anti-TNF antibodies should be considered as first line therapy for metastatic Crohn´s disease.

In case of treatment failure, there are less common therapeutic options such as ustekinumab, surgical intervention or hyperbaric oxygen therapy, which can be offered to the patient. Although the efficacy cannot be evaluated based on the limited amount of available data, the existing literature as well as our case report suggests a positive effect of ustekinumab in patients with metastatic Crohn’s disease.

Topical tacrolimus can improve the lesions, but there is no case report where topical tacrolimus could induce remission [3, 5, 9, 24, 39, 57, 61], and can currently not be recommended as a single therapeutic option. It might be useful to support other therapies.

## Conclusion

Metastatic Crohn’s disease can affect patients with or without GI involvement. Diagnostics include anamnesis, inspection and biopsies of the involved site as well as endoscopy and MRI.

Currently, with no German, European or American guidelines available, approved therapies for intestinal Crohn’s disease are employed, based on the suspected similar inflammatory pathophysiology in intestinal and extraintestinal sites: Steroids, anti-TNF antibodies, and antibiotics were the primary and most potent agents. Mesalazine and azathioprine as well as less common treatment options such as surgical intervention or hyperbaric oxygen therapy may also be considered in case of treatment failure.

With the increasing number of reported cases of metastatic Crohn’s disease, the need for future guidelines for treating these patients becomes apparent. Our case demonstrates successful ustekinumab treatment for metastatic Crohn’s disease, suggesting a potential new therapeutic option.

## Data Availability

Laboratory results, histological findings, imaging and other diagnostic results are available if necessary.
